# Cutaneous Squamous Cell Carcinoma with Invasion through Ear Cartilage

**DOI:** 10.1155/2016/9067428

**Published:** 2016-05-16

**Authors:** Julie Boisen, C. Helen Malone, Brent Kelly, Richard F. Wagner

**Affiliations:** Department of Dermatology, University of Texas Medical Branch, Galveston, TX 77550, USA

## Abstract

Cutaneous squamous cell carcinoma of the ear represents a high-risk tumor location with an increased risk of metastasis and local tissue invasion. However, it is uncommon for these cancers to invade through nearby cartilage. Cartilage invasion is facilitated by matrix metalloproteases, specifically collagenase 3. We present the unusual case of a 76-year-old man with an auricular squamous cell carcinoma that exhibited full-thickness perforation of the scapha cartilage. Permanent sections through the eroded cartilage confirmed tumor invasion extending to the posterior ear skin.

## 1. Introduction 

Cutaneous squamous cell carcinoma (cSCC) is the second most common cancer of the skin in the United States. The ears are a frequent site of these cancers and represent a high-risk location with an increased risk of metastasis. Invasion of cartilage by cSCC is rare and considered to be a significant risk factor for the development of metastatic disease. Current treatment guidelines recommend surgical excision with histologic confirmation of negative margins and close follow-up to monitor for recurrence and metastasis. We present the case of a 76-year-old man with a cSCC on his anterior left ear with tumor perforation through his auricular cartilage that permitted tumor spreading to posterior ear skin.

## 2. Case Presentation 

**Figure 1:**
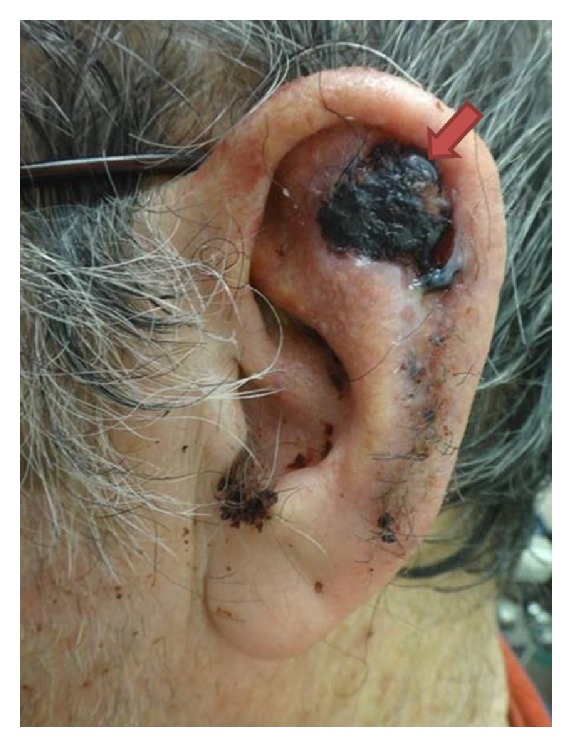
76-year-old Caucasian man with a moderately differentiated squamous cell carcinoma covered by hemorrhagic crust on the left anterior upper ear (arrow).

A 76-year-old Caucasian man with advanced dementia who was anticoagulated daily with 81 mg of aspirin and 75 mg of clopidogrel due to a history of cerebrovascular accident presented to the dermatology department with a biopsy proven moderately differentiated squamous cell carcinoma on the left ear. The tumor measured 2 by 2.7 cm, involved the left scapha, and clinically extended to the superior crus of the antihelix (Figure 1). It was symptomatic for associated pain and bleeding. He did not have any palpable lymphadenopathy in the head and neck region; therefore further investigative techniques such as imaging and sentinel lymph node biopsy were deferred. The diagnostic biopsy was performed using a tangential shave technique that did not involve cartilage and the tumor was subsequently staged as T2N0M0 according to the American Joint Committee on Cancer staging system. After an extensive discussion with the patient's daughter and taking into consideration his multiple comorbid conditions including a history of cerebrovascular accident with resultant hemiplegia, hypertension, COPD, asthma, borderline diabetes mellitus, hepatitis B virus, dyslipidemia, and dementia, Mohs micrographic surgery (MMS) was selected as the optimal treatment modality.

**Figure 2:**
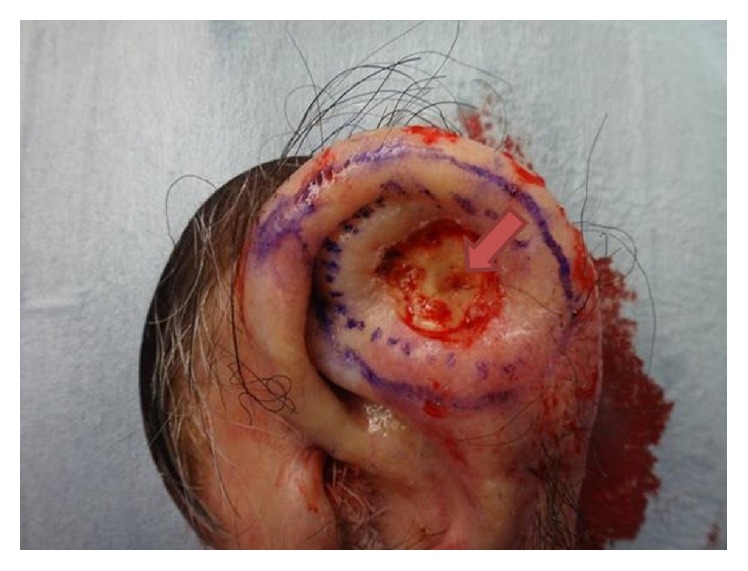
Left ear status after curettage of grossly positive tumor exposing perforation of scapha cartilage (arrow).

**Figure 3:**
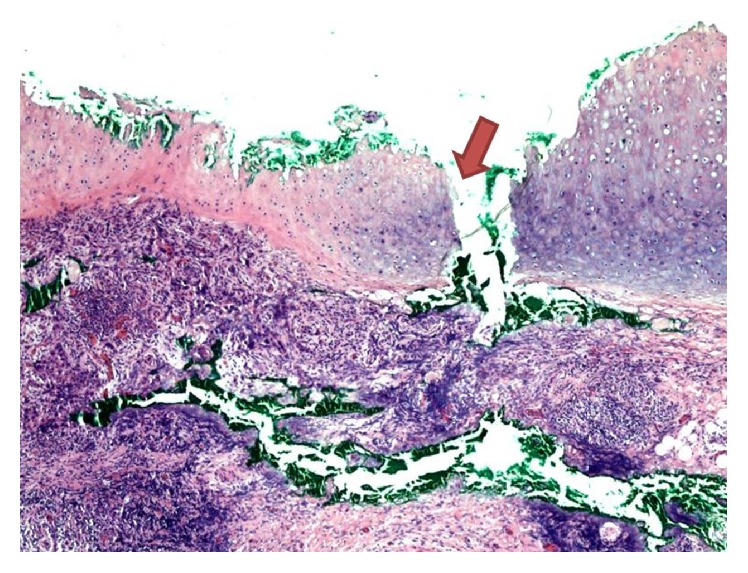
40x magnification of a permanent section taken through the perforated cartilage (arrow). The anterior skin above the cartilage is absent (status after curettage) and the posterior perichondrium and dermis are seen inferior to the cartilage.

**Figure 4:**
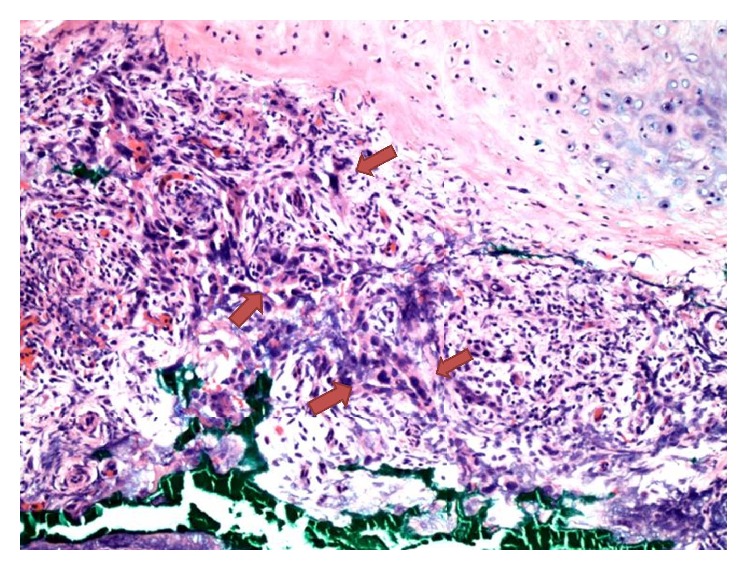
100x magnification of area directly below the perforated cartilage. Large pleomorphic cells are present (arrows).

**Figure 5:**
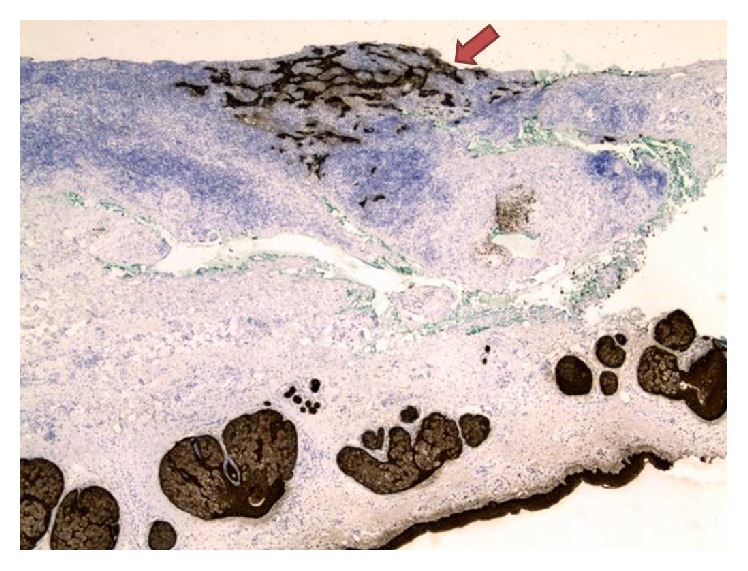
20x magnification of tissue taken directly below the perforated cartilage (cartilage removed during immunostaining). Large pleomorphic cells staining positively for AE1/AE3 (arrow) are present in the posterior ear skin. The normal pilosebaceous units, eccrine ducts, and epidermis in the normal posterior ear skin serve as positive controls.

After a surgical site preparation and infiltration of 1% buffered lidocaine with epinephrine 1 : 400,000, a curette was used to gently debride the clinical tumor. A 2 mm full-thickness defect in the cartilage of the scapha was noted (Figure 2). Due to the violation of the cartilaginous barrier by the patient's tumor, the posterior skin was included in the resection because of increased risk of tumor spreading through the cartilage. Negative margins were achieved with I Mohs stage. The option for a more aesthetic reconstruction was declined, so the wound was closed primarily with 3-0 chromic suture. The area of cartilage perforation was sent for permanent processing to confirm invasion of the tumor through the cartilage (Figures 3 and 4). Additional staining with AE1/AE3 was positive, further confirming the diagnosis of cSCC in the posterior ear skin with full-thickness cartilage perforation (Figure 5).

## 3. Discussion 

Although the classification of high-risk cSCC is not uniform among dermatologists, most agree on a few key clinical and histopathologic features. High-risk clinical features include clinical size ≥2 cm, involvement of the lip, ear, or mid face, poorly defined clinical borders, neurologic symptoms, tumor destruction of cartilage, immunocompromised host, recurrent or rapidly growing tumor, and tumor arising in a scar, chronic ulcer, or site of prior radiotherapy. The histopathologic elements that are associated with increased tumor risk include depth >4 mm, moderate or poor cytologic differentiation, adenoid, acantholytic, adenosquamous or desmoplastic subtypes, and perineural or lymphovascular invasion [1]. Poorly differentiated cSCC stains more strongly for AE1/AE3 compared to well-differentiated neoplasms and generally indicates a poorer prognosis [2]. Based on the findings from these studies, this patient's tumor is considered high-risk due to a clinical diameter greater than 2 cm, location on the ear, moderate to poor differentiation, and invasion of cartilage.

Matrix metalloproteases (MMP) play an integral role in tumor growth and metastasis. MMPs are a family of zinc-dependent endopeptidases that function to remodel the extracellular matrix (ECM). They allow tumors to grow by degrading matrix barriers and promoting angiogenesis as well as releasing active growth factors and modulating apoptosis. Direct invasion of local structures by tumors is facilitated by MMP-mediated proteolytic degradation of the ECM. Markers of malignant transformation of keratinocytes include MMP-13, MMP-7, MMP-12, and MMP-14.

Specifically, MMP-13 is associated with greater metastatic capacity and MMP-11 is linked to increased local invasiveness of SCC of the head and neck [3]. MMP-13 (collagenase 3) preferentially degrades Type II collagen found in cartilage and is expressed in malignant keratinocytes found in cSCC. Normally laminin-5, which is found in the basement membrane, promotes keratinocyte motility. In cSCC, MMP-13 colocates with laminin-5 to the edge of lesion and subsequently degrades nearby tissue, allowing tumor invasion [4].

Cutaneous squamous cell carcinoma with auricular involvement has a metastatic rate of approximately 15.5% and destruction of cartilage is a significant risk factor for metastatic disease [5–7]. The most commonly affected lymph nodes include the parotid, submandibular, submental, and superior cervical nodes. Lymph node palpation has a sensitivity of 60 to 75% for detecting lymph node metastasis [1]. Tumor depth and diameter are the most important prognostic factors for determining risk of local recurrence and metastasis [6]. Tumor depth could not be measured in this patient due to treatment with MMS that included curettage to debulk gross tumor to define its extent prior to excision and en face horizontal frozen sections for tumor extirpation. Patients with high-risk cSCC and suspected regional metastasis may benefit from elective neck dissection or sentinel lymph node biopsy to detect subclinical nodal involvement; however this remains controversial.

Current guidelines by the National Comprehensive Cancer Network (NCCN) for high-risk cSCC advocate surgical excision by either standard excision with postoperative margin assessment or MMS. Primary radiation therapy is also an option for nonsurgical candidates. MMS results in a 5-year disease-free survival rate of 97% for patients with cSCC [8]. Postoperative radiation therapy reduces the rate of recurrence in high-risk patients and is recommended for patients with substantial perineural involvement or in cases where negative margins cannot be achieved [8]. This patient and his family declined adjuvant radiation therapy, and they plan for regular follow-up visits.

## 4. Conclusion 

High-risk features for cSCC include clinical tumor size ≥2 cm, tumor depth >4 mm, moderate to poor histologic grade, high-risk anatomic location, perineural involvement, and invasion of cartilage. Treatment consists of surgical excision with adjuvant radiation therapy in the setting of large nerve perineural invasion by cSCC, other high-risk tumor features, or inability to obtain negative margins. The role of sentinel lymph node biopsy and elective neck dissection in treatment of cutaneous squamous cell carcinoma remains controversial and without current proven clinical efficacy. This case report represents a pathologically confirmed observation of an anteriorly positioned primary cutaneous squamous cell carcinoma with full-thickness auricular cartilage perforation leading into tumor invasion of the posterior ear skin.
